# Studies on the weight of the gastrointestinal tract, digesta composition and occurrence of gastro- and enteroliths in adult domesticated ostriches fed different diets

**DOI:** 10.1016/j.psj.2021.101359

**Published:** 2021-06-26

**Authors:** Amr Abd El-Wahab, Frederick Fimmo Schuchmann, Bussarakam Chuppava, Christian Visscher, Christiane Pfarrer, Josef Kamphues

**Affiliations:** ⁎ Department of Nutrition and Nutritional Deficiency Diseases, Faculty of Veterinary Medicine, Mansoura University, 35516 Mansoura, Egypt; † Institute for Animal Nutrition, University of Veterinary Medicine Hannover, Foundation, D-30173 Hannover, Germany; ‡ Institute for Anatomy, University of Veterinary Medicine Hannover, Foundation, D-30173 Hannover, Germany

**Keywords:** ostrich, diet, digesta, stone

## Abstract

Comminuting the ingested material in the stomach and fermentation in the large intestine of ostriches, allows an efficient utilization of fiber-rich feedstuffs. The entire gastrointestinal tract (**GIT**) of 61 adult ostriches (both sexes; av. age of 15 mo), which had previously been fed a ration consisting of either haylage and pelleted compound feed (**HP**) or haylage, corn silage and pelleted compound feed (**HCP**), was the subject of the present investigations. The weight of the different compartments of the GIT was measured. The digesta was differentiated into inorganic and organic substances. Wet sieving was used to separate the collected stones (>19 mm), small stones (1 mm), and sand (<1 mm). Ostriches fed the HCP diet had a significantly higher empty gizzard weight (3,435 g) compared to those fed the HP diet (3,064 g). Additionally, the weight of the empty cecum (left and right parts) was increased (*P* < 0.05) for ostriches fed the HCP diet (107 and 122 g, respectively) in comparison to those fed the HP diet (93.4 and 108 g, respectively). The weight of pure digesta in the gizzard and left or right cecum for ostriches fed the HP diet was higher (1,640, 448, and 471 g, respectively) compared to those fed the HCP diet (*P* < 0.05). The contents of crude ash and HCl-insoluble ash in the digesta of all the GIT compartments were higher for ostriches fed the HP diet in comparison to those fed the HCP diet (*P* < 0.05). Independent of the type of the offered diet, the large stones occurred only in the proventriculus and gizzard (2.71 and 4.76%, respectively), while sand dominated in the distal colon (30.3%). The high proportion of stones in the gizzard form the “mechanical equipment” which enables the animals to grind basic feed such as corn silage or haylage, and these are almost completely excreted as sand. Continuous stone replacement for ostriches is necessary but the amount mostly depends on the type of feed.

## INTRODUCTION

Ostriches (*Struthio camelus*) are the largest recent species in birds and belong to the group of ratites along with the nandus and emus (Cooper et al., 2001). The first commercial ostrich farm was established in South Africa in about 1860 for obtaining feathers (Shanawany, 1999). Ostrich farms began to spread gradually to other countries, particularly Egypt, Australia, New Zealand, the United States, and Argentina (Shanawany, 1999). This made ostrich farming an attractive proposition, and first farms were also established in Europe and even more so in the United States to fill part of the increasing global demand for feathers, skin and meat (Shanawany, 1999; Kistner, 2019).

Ostriches share many of the evolutionary adaptations of other birds, but some features, such as the gastrointestinal tract (**GIT**) morphology, are unique to the families of ratites, for example, the absence of a goiter (Bruning and Dolensek, 1986). Additionally, ostriches have deep proventricular glands restricted to a slipper-shaped area, these extending to the muscularis mucosae; while the gizzard exhibits a variably developed muscularis mucosae (Bezuidenhout and van Aswegen, 1990). The increased thickness of the muscularis mucosa and muscular layer of the proventriculus and gizzard leads to stronger muscle rhythm and enhanced digestive ability (Wang et al., 2017).

With the help of ingested stones and pebbles, together with the contractions of the muscular walls, the ingested plant material is mechanically finely ground – similar to a ball mill – (Holtzhausen and Kotzé, 1990; Wings and Sander, 2007). The isthmus between the proventriculus and the gizzard has a relatively wide lumen, which allows the passage of bulky digesta and stones (grinding processes), while the pylorus is very narrow, allowing only the passage of ground feed and smaller stones/sand (Huchzermeyer, 1998). Nickel et al. (2004) described the process of grinding the feed structure in the gizzard, which is similar to the principle of a grist mill, because the muscles of the filled gizzard exert high pressure on the stomach contents through longitudinal contractions and rotary movements. At the same time, the digestion of the feed is promoted by the strongly acidic environment (pH 2.2) in the muscular stomach (Holtzhausen and Kotzé, 1990). The small stones in the gizzard help the gradual mechanical breakdown of feed, which then passes through the gizzard (Miao et al., 2003).

The fermentative degradation of crushed plant fibers primarily depends on the microflora and the retention time of the digesta in the fermentation chamber (Sales, 2006). The retention time of the digestion components in birds particularly depends on the particle size (Dziuk and Duke, 1972; Clemens et al., 1975). An efficient digestion of plant fiber substances requires a rather slow ingesta flow rate and an area in the digestive tract where microbes can settle and multiply without being carried along by the intestinal passage (Deeming, 1999). Ostriches have the most efficient postgastric/hindgut fermentation of plant fibers among birds and are therefore more comparable to horses or rabbits in their digestive physiology (Skadhauge et al., 1996; Kistner and Reiner, 2002). Furthermore, the long retention time of fibrous feed in the GIT ensures exposure of feed particles to microbial digestion for extended periods (Swart et al., 1993). In the course of its growth, the ostrich also develops the ability to utilize crude fiber and, already in the 10th week of life is able to live on a feed consisting of 50% roughage as haylage and corn silage (Kistner and Reiner, 2002). During the microbial digestion of crude fiber, volatile fatty acids are produced in the fermentation tract (ceca, cranial section of the colon) of the ostrich. Ostriches can obtain up to 76% of their required energy in the form of free volatile fatty acids (mostly acetate) from plant fibers (Swart et al., 1993).

The term “gastrolith” was first defined and scientifically used by Mayne (1860): “Gastrolith - a stone in the stomach”. Wings (2004) further claims that all stones found in the digestive tract should be called “gastroliths”. If no stones are available, the animals can even die of digesta compaction in the stomach (Huchzermeyer, 1994; Wings and Sander, 2007). Therefore, this study aimed to investigate the effects of feeding different diets (haylage or haylage + corn silage) containing different fiber contents on the masses (organs with/without digesta) as well as the contents (e.g., digesta composition or stones/pebbles) of the individual stomach compartments. Finally, this study should provide a deeper understanding concerning the need for a continuous uptake of stones by ostriches and their fate and size distribution during the GIT passage.

## MATERIALS AND METHODS

In total, organs from 61 ostriches were obtained at slaughtering. Since no interventions had been carried out on live animals, the study was not an animal experiment according to the Animal Protection Act, and thus did not require approval from the respective authority.

### Housing and Feeding

**Figure 1:**
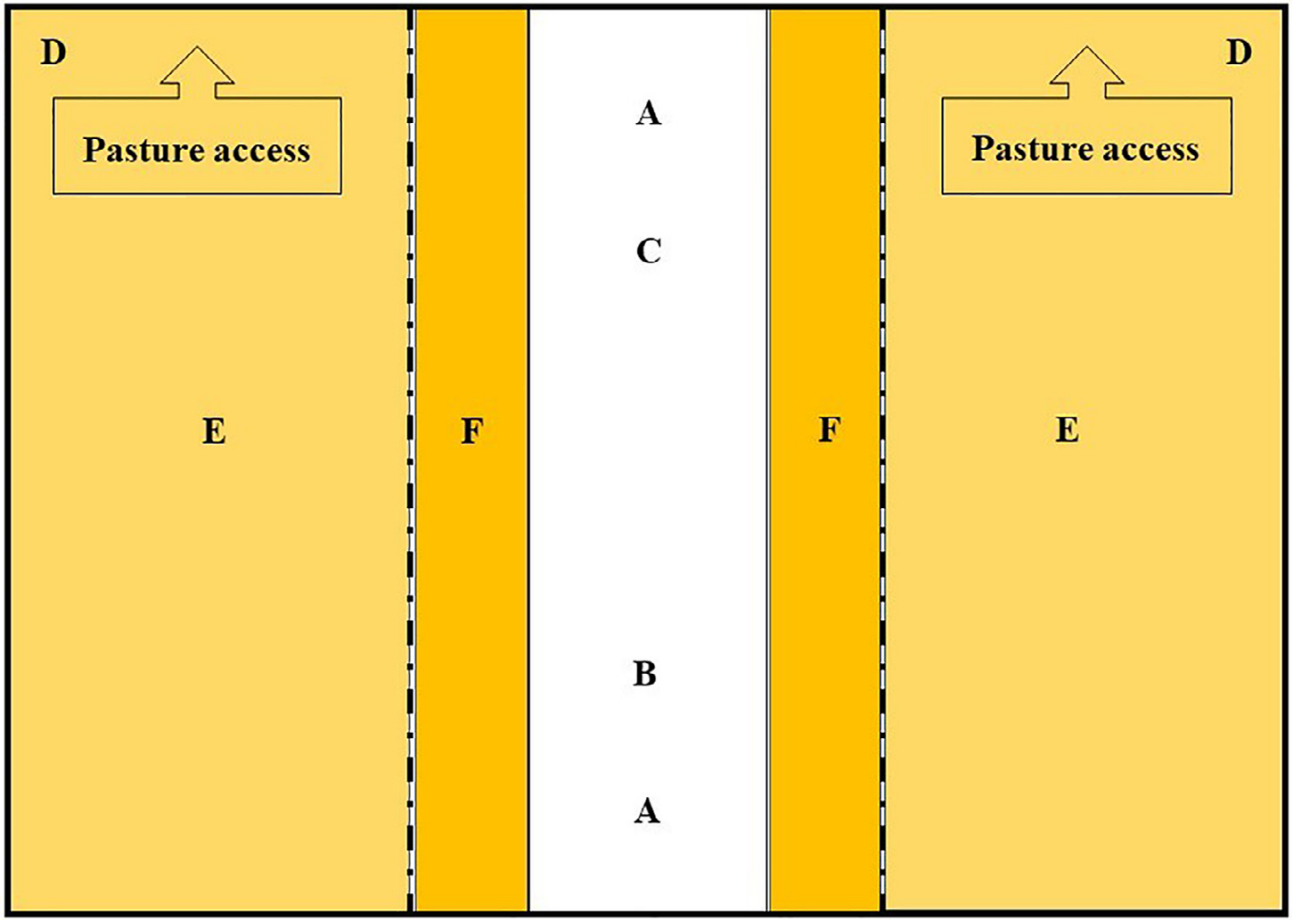
Construction of a barn for adult animals; A, compound feed container, B, hay bales, C = corn silage bales, D, water trough, E, bedding area, F, feeding area.

The ostrich farm was located in North Rhine-Westphalia, Germany. The entire farm comprised an area for the breeding groups with 12 enclosures. The barn for adult ostriches was constructed with a feeding and drinking area (Figure 1) as well as a bedding area littered with straw. However, the covered entrance for the animals was littered with sand. Furthermore, the sand served as an additional comfort zone, for example, sand bathing for all animals.

**Figure 2:**
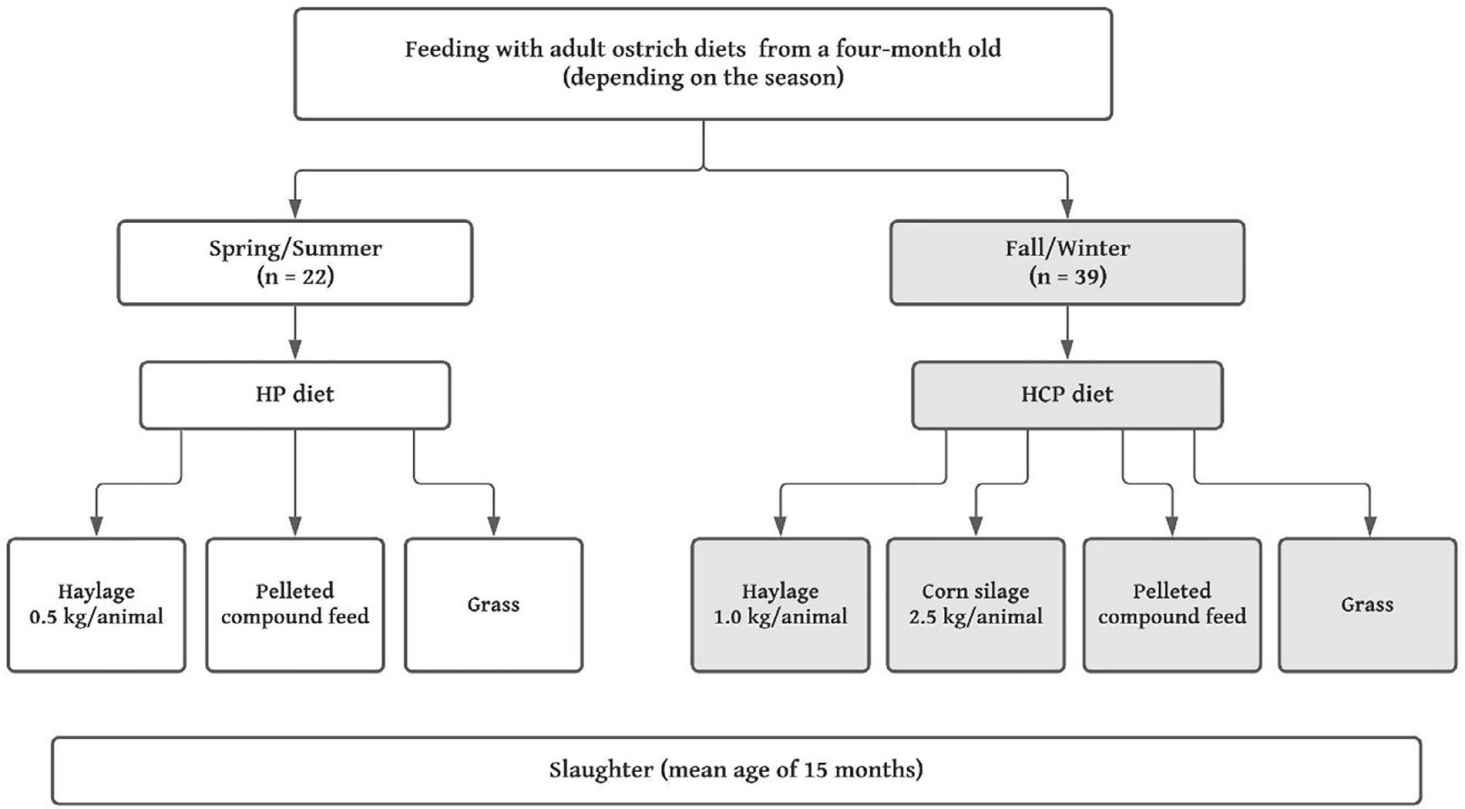
Feeding scheme for the adult ostriches in this study.

Before slaughter, the 61 animals (33♂; 28♀) had free access to grazing areas throughout the year. Depending on the season and the amount of grazing areas, the ostriches were provided with additional feed twice a day (Figure 2). In spring/summer, the animals (12♂; 10♀; total = 22) were fed a ration of about 0.5 kg haylage and about 0.7 kg pelleted compound feed/animal. This group was called the HP group. In the fall/winter, the animals (21♂; 18♀; total = 39) were fed a ration of haylage (about 1.0 kg/animal) and whole plant corn silage (about 2.5 kg/animal) as well as pelleted compound feed (about 1.0 kg/animal). These birds were referred to as the HCP group. These 2 rations, as supplement to grazing, are very common in Germany for feeding ostriches, thus this study focused on using these feeding systems. Also, it has to be mention that in the current study, each bird in each group was considered as an experimental unit.

Groups were slaughtered at the mean age of 15 mo. The pelleted compound feed was provided by Eilers Futtermittel GmbH & Co. KG, Saerbeck, Germany. It was composed of corn, barley, wheat, wheat bran, and soybean meal (Table 1). The analyzed composition of the feeds used in this study is shown in Tables 1 and 2. Stones up to 70 mm were offered to adult ostriches in all enclosures. However, it has to be mentioned that pebbles of 4 to 40 mm were offered to ostrich chicks from the first day of life.

**Table 1:** Ingredient composition of the compound feed for adult ostriches.

Ingredient	%
Corn	15.0
Barley	13.0
Corn flakes	13.0
Wheat	7.00
Wheat bran	19.0
Soybean meal	21.0
Sunflower oil	3.00
Mineral feed^1^	9.00
Feed chemical composition (%)	
Crude Protein	16.8
Crude fiber	5.90
Crude ash	9.30
Ether extract	5.82
Calcium	2.10
Phosphorus	1.00
Sodium	0.30
Lysine	0.84
Methionine	0.50
Additives per kg as fed	
Vitamin A, IU	26620
Vitamin D, IU	4437
Vitamin E, mg	111
Copper, mg	19.2
Selenium, mg	0.71

1 Produced by SALVANA Tiernahrung GmbH, Ahlhorn, Germany and contained: calcium carbonate, sodium chloride, mono-calcium phosphate and magnesium oxide (in %, DL-Methionine, 2.60; Calcium, 23.5; Phosphorus, 7.50; Sodium, 3.50; Magnesium, 0.37).

**Table 2:** The contents of dry matter, organic and inorganic materials, the pH-value and particle size distribution (%) in haylage, corn silage and pelleted compound feed.

Parameter (% DM)	Haylage	Corn silage	Pelleted compound feed
Dry matter (as fed)	70.9	31.0	82.7
Crude protein	12.9	7.42	20.7
Crude fiber	29.0	19.9	5.95
Crude ash	12.5	3.18	6.60
HCl-unsoluble ash	5.34	0.61	0.68
pH value	5.70	3.88	6.30
Particle size (%)			
>3.15 mm	77.4 ± 5.55	62.4 ± 0.59	1.63 ± 1.81
2.0 mm	0.95 ± 0.28	4.61 ± 0.68	4.78 ± 3.32
1.4 mm	0.91 ± 0.15	2.25 ± 0.23	11.8 ± 1.22
1.0 mm	0.61 ± 0.34	1.91 ± 0.55	15.7 ± 1.88
0.8 mm	0.35 ± 0.21	0.94 ± 0.42	8.16 ± 0.06
0.56 mm	0.38 ± 0.23	1.41 ± 0.53	11.5 ± 1.68
0.4 mm	0.49 ± 0.16	0.91 ± 0.75	7.35 ± 1.29
0.2 mm	0.46 ± 0.17	0.74 ± 0.85	8.28 ± 2.43
<0.2 mm	18.4 ± 5.61	24.8 ± 3.62	30.8 ± 2.90

The particle size distribution (%) is shown in means ± SD.

### Dissection

**Figure 3:**
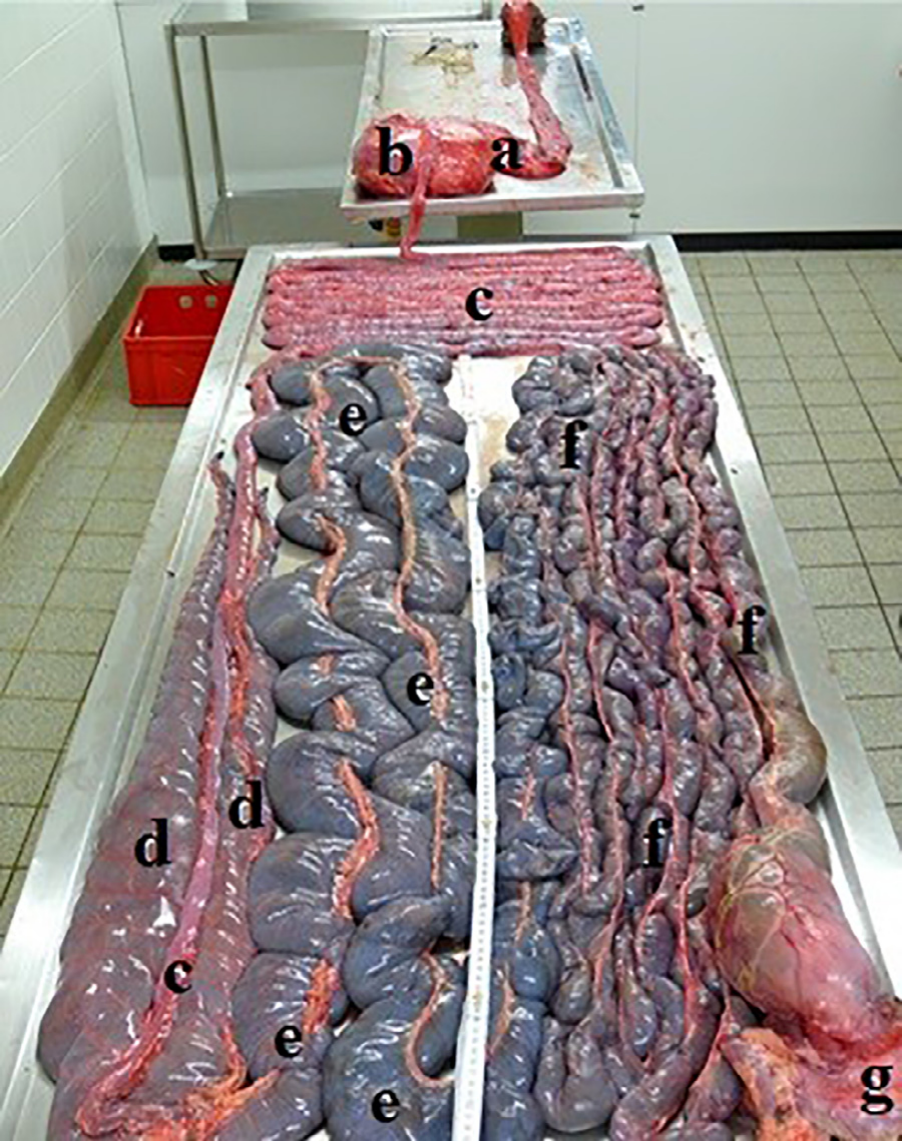
Gastrointestinal tract of an ostrich; A, glandular stomach, B, gizzard, C, small intestine, D, ceca, E, proximal / F, distall colon section, g, cloaca.

Until transport to the slaughterhouse (duration of transport: 1 h), the animals received a mixture of the abovementioned feedstuffs and water ad libitum. About 8 ostriches were slaughtered per month in terms of commercial slaughtering, 22 during spring and summer, and 39 during fall and winter. In a stand designed for ostriches, stunning was performed by means of electric tongs (Type K 300, JWE-Baumann GmbH, Aalen-Oberalflingen, Germany). After death, the body mass was determined. The whole intestine and the internal organs were removed from the body cavity (Figure 3). The digestive tract was stored at +4°C up to the end of the slaughtering process, cool-transported to the university (about 3 h) and stored up to further processing at −18°C. The weight of the GIT sections was determined again after emptying.

#### Chemical Analyses of Feed and Digesta

The feed materials and the digesta of different sections of the GIT were subjected to chemical analysis based on the official methods of the Association of German Agricultural Analytic and Research Institutes (**VDLUFA**) in accordance with Naumann and Bassler (2012). The dry matter (**DM**) content was calculated by weighing before and after drying the samples at 103°C. The Dumas incineration method (Vario Max, Elementar, Analysensysteme GmbH, Langenselbold, Germany) was applied to measure the total N content of the feeds. The muffle furnace was used to determine the crude ash content by weighing the samples before and after combustion at 600°C. The crude fiber content was determined by washing the samples in diluted acids and alkalis. For analyzing the crude fiber and inorganic contents (crude ash and HCl-insoluble ash), the respective feed and the digesta of the individual sections of the GIT were freeze-dried and ground (Centrifugal Mill ZM 1000, Retsch GmbH, Haan, Germany).

#### pH Value of Digesta

The pH values in the digesta obtained from the duodenum, jejunum, ileum, and the 2 ceca were determined directly after their collection. A certain amount of the digesta was diluted in a ratio of 1:4 with distilled water and remained for 30 min at room temperature. The pH value was determined using a calibrated glass electrode (HI 2211 pH/ORP Meter, Hanna Instruments Deutschland GmbH, Vöhringen, Germany).

#### Fractionation of the Gastro- and Enteroliths

**Figure 4:**
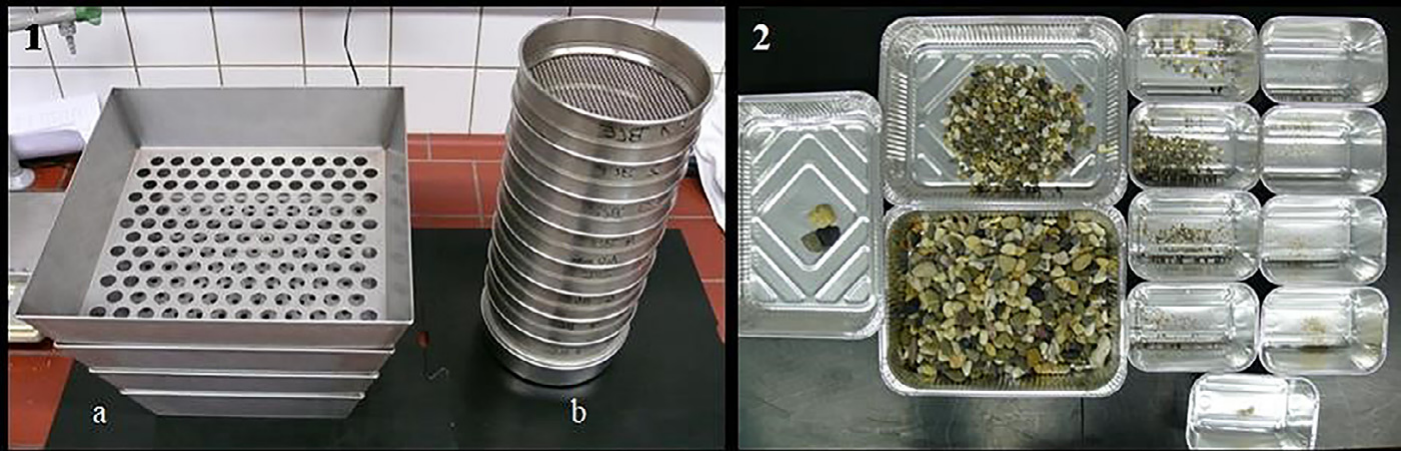
Fractionation of the gastro- and enterolith mixtures by means of a shaking box (1a) and a sieve tower (1b); gastroliths from the contents of a gizzard (2) divided into 12 fractions.

The separation of the gastrolith and enterolith mass was carried out in 2 successive steps, namely rinsing and decanting. The contents of each section were rinsed with water until pure sediment of stones, pebbles or sand remained on the bottom of the vessel. These mixtures were rinsed with distilled water and dried at 103°C for at least 24 h until their weight remained constant. By means of a shaker box as shown in Figure 4 (Shaky 4.0; Wasserbauer GmbH, Waldneukirchen, Austria; 19 / 8 / 4 mm) and a sieve tower (Retsch GmbH; 3.15 / 2.0 / 1.4 / 1.0 / 0.8 / 0.56 / 0.4 / 0.2 mm) with a total of 11 sieve elements of different mesh sizes. In the following sections, the fractions >19 mm were defined as “stones”, the fractions between <19 mm and >1 mm were referred to as “pebbles” and the remaining fractions <1 mm were defined as “sand”.

#### Particle Size Distribution in Feed and Pure Digesta

Using wet sieve analysis, the particle size distribution of the different feeds and the chyme of the GIT (glandular stomach, gizzard, ileum, pooled ceca, proximal and distal sections of the colon) were determined. Depending on the amount of chyme material, 50 to 200 g were weighed and mixed with distilled water (800 mL). Thereafter, the samples were left to stand for 30 min and carefully mixed 1 to 2 times. Any sediment, consisting of gastro- or enterolites, depending on the sample material, was rinsed after pouring off the supernatant, dried at 103°C for at least 24 h and the weight was subtracted from the weight of the sample material.

These gastro- or enteroliths were recorded in the dry sieve analysis of the stones. The suspensions were each sieved through a stainless-steel sieve tower (Retsch GmbH) with 8 individual sieves of different mesh sizes (3.15/2.0/1.4/1.0/0.8/0.56/0.4/0.2 mm, respectively). The sample material was rinsed with 10 L of distilled water evenly over the entire surface of the sieve. The towers were dried to a constant weight at 103°C and the weight of the individual sieves was determined.

### Statistical Analysis

The statistical analysis of the results was carried out in cooperation with the Institute of Biometry, Epidemiology and Information Processing, University of Veterinary Medicine Hannover, Foundation, Hannover, Germany. The software used for data processing was SAS Enterprise Guide Version 7.1 (Cary, NC). For quantitative characteristics, the data were first checked for normal distribution (Shapiro-Wilk test). Normally distributed data were analyzed for the smallest significant differences using a simple analysis of variance (Fisher's Test). To verify differences in non-normally distributed data, a pairwise comparison with the Wilcoxon test was performed. The statistical significance was considered at *P* < 0.05.

## RESULTS AND DISCUSSION

The present study focused on the impacts of feeding different dietary fiber contents on the GIT masses and digesta components as well as on the “fate” of the stones.

### Composition of the Diet

In addition to grazing, the feed ingredients in this study depended on the season, for example, the HCP diet (haylage, pelleted compound feed, and corn silage) was given in the fall and winter, while in spring and summer, the ostriches were fed with the HP diet (haylage and pelleted compound feed). The composition and particle size distribution of haylage, corn silage, and pelleted compound feed are shown in Table 2. The level of crude fiber in haylage was about 29.0 vs. 19.9% for corn silage. The chemical composition of the plants selected by the ostriches was about 11.2% protein, 4.2% lipids, and 35.2% crude fiber on DM basis (van Niekerk, 1995). Milton et al. (1994) found that ostriches feeding on natural forage required a diet containing about 24% fiber, 12% crude protein, 16% ash, and 3% lipids on a DM basis, for maintenance. Regarding the particle size distribution, the haylage had the highest fraction of 3.15 mm (77.4%) and the lowest fraction of <0.2 mm (18.4%), while the corn silage had about 62.4 and 24.8% particle size distribution for >3.15 mm and <0.2 mm, respectively. It has to be underlined that due to the semi-extensive feeding of the ostriches, there was no possibility to determine the actual mass of feed consumed, as the rations were not offered as a total mixed ration.

### Body Weight and GIT Measurements

The mean body weight (**BW**) of adult ostriches (means of 15-mo-old ostriches at slaughter) was about 105 kg ± 10.0 and 108 kg ± 11.2 for those groups fed the HCP and HP diets, respectively. Kistner and Reiner (2002) mentioned that BW for domesticated African ostriches was between 110 and 150 kg. Moreover, Miao et al. (2003) suggested that the differences in BW of ostriches are partially a result of the diet quality rather than the age of slaughter. For example, ostriches in South Africa are normally slaughtered at 14 mo of age at a weight of 95 to 110 kg; while in United States, BW of 120 to 130 kg is common in 12-mo-old because in the United States, diets tend to be of higher quality and rearing tends to be intensive rather than extensive as in South Africa (Miao et al., 2003).

The duodenum of ostriches in the HCP feeding group was 4.70% longer than the duodenum of animals in the HP feeding group (*P* < 0.05). However, no significant differences were observed in the length of either the jejunum or the ileum between both groups (details in Table S1). The weight of the empty gizzard and cecum (left and right parts) of ostriches as well as the weight of pure digesta are presented in Table 3. Ostriches fed the HCP diet had a significantly higher weight of empty gizzard and both left and right cecum (3,435, 107, and 122 g, respectively) in comparison to those fed HP. This means that the ratio of the relatively empty gizzard weight to body weight was about 3.27 and 2.84% for groups fed the HCP and HP diets, respectively. In contrast, the weight of pure digesta in the gizzard and both the left and right cecum (1,640, 448, and 471 g, respectively) was higher in the group fed the HP diet (*P* < 0.05) compared to those animals fed the HCP diet. The weight of pure digesta in the gizzard in relation to the body weight was about 0.84 and 1.52% for groups fed the HCP and HP diets, respectively. The heaviest organ weight of the GIT was the gizzard even with or without digesta. Klasing (1998) observed that the muscular gizzard of the herbivorous birds that eat soft and easily digestible diet are very similar to the glandular stomach in thickness and muscular development. Depending on the diet, the size of the gizzard is pronounced (Piersma et al., 1993). Herbivorous species, such as ratites or geese, which eat rations with a high proportion of grass and leaves, have greatly enlarged muscular stomach muscles in contrast to species fed on ground cereals (Klasing, 1998). The high amount of pure digesta in the gizzard and cecum in ostriches fed the HP diet may indicate more fermentation of crude fiber. In our study, it has to be mention that, in general, the grasses grow much higher in spring/summer season in Germany compared to fall/winter season. Consequently, it seems that ostriches in HP group fed more fresh grasses than those in HCP group. Moreover, according to Ates and Tekeli (2005) Orchardgrass (*Dactylis glomerata*) is one of the most productive cool-season grasses, fairly drought resistant, and is widely distributed with crude fiber content of about 23.8%. It is well know that the 2 of the most prominent factors affecting digestion efficiency of nutrients in the presence of soluble fiber are solubility and fermentability because of their impact on passage rate in the small intestine and the fermentability in the hindgut, respectively (Kheravii et al., 2018). Soluble fibrous components of the diet such as pectins and arabinoxylans have been regarded to increase intestinal viscosity, reducing the absorption of nutrients and modulating digesta passage rate (Mateos et al., 2012). Swart et al. (1993) concluded that the long retention time of fibrous feed and/or the digesta in the GIT ensures exposure of feed particles to microbial digestion for extended periods and a high concentration of volatile fatty acids. The movement of the digesta out of the gizzard is based on particle size, which is controlled by the small openings of the pylorus, which functions as a sieve (Svihus, 2011). The larger particles of dietary fiber will help in the retention of bolus in the upper portion of the GIT, slowing down the passage rate and increasing the exposure of feed components to HCl and enzymes from the proventriculus. This results in the accumulation of insoluble fiber in the gizzard and increases digestibility of nutrients (Sacranie et al., 2012).

**Table 3:** Influence of different feeding types on the body weight, weight of empty gizzard, and cecum as well as on pure digesta.

Item	Weight, g	HP	HCP	*P*-value
Body weight, kg		108^a^ ± 2.38	105^a^ ± 1.61	0.257
Empty organ	Gizzard	3064^b^ ± 110	3435^a^ ± 88.2	0.012
	Cecum, left part	93.4^b^ ± 2.86	107^a^ ± 3.40	0.011
	Cecum, right part	108^b^ ± 2.79	122^a^ ± 4.38	0.022
Digesta	Gizzard	1640^a^ ± 157	885^b^ ± 96.7	0.039
	Cecum, left part	448^a^ ± 44.4	331^b^ ± 27.2	0.014
	Cecum, right part	471^a^ ± 43.3	336^b^ ± 30.6	0.012

Abbreviations: HP, Haylage+Pellet compound feed (n = 22); HCP, Haylage+Corn silage+Pellet compound feed (n =39).

a,b Different superscripts within row mark significant differences between the groups (*P* < 0.05).

### Digesta Composition

The DM and crude fiber contents of the digesta in the individual sections of GIT are shown in Table 4. The DM content of digesta in the proventriculus and gizzard were significantly higher for ostriches fed the HP diet (222 and 330 g/kg DM, respectively) than for those animals fed the HCP diet (174 and 242 g/kg DM, respectively). Ostriches fed the HCP diet had a significantly higher crude fiber content in the digesta of the proventriculus and gizzard (240 and 247 g/kg DM, respectively) than those fed the HP diet (205 and 147 g/kg DM, respectively). Also, the crude fiber content was significantly higher in the digesta of the cecum (pooled) and proximal and distal parts of the colon (131, 184, and 212 g/kg DM, respectively) compared to those animals fed the HP diet (86.8, 89.6, and 95.3 g/kg DM, respectively).

**Table 4:** Influence of diets on the dry matter and crude fiber contents in the GIT digesta of ostriches.

Parameter(g/kg DM)	Organ	HP	HCP	*P*-value
Dry matter	Proventriculus	222^a^ ± 11.4 (n = 20)	174^b^ ± 6.59 (n = 38)	<0.001
	Gizzard	330^a^ ± 17.5 (n = 21)	242^b^ ± 6.59 (n = 39)	<0.001

Crude fiber	Proventriculus	205^b^ ± 13.0 (n = 20)	240^a^ ± 9.15 (n = 32)	0.026
	Gizzard	147^b^ ± 9.11 (n = 20)	247^a^±6.94 (n = 36)	<0.001
	Cecum (pooled)	86.8^b^ ± 8.28 (n = 20)	131^a^ ± 6.64 (n = 32)	<0.001
	Colon (proximal)	89.6^b^ ± 7.10 (n = 20)	184^a^ ± 4.92 (n = 39)	<0.001
	Colon (distal)	95.3^b^ ± 9.14 (n = 20)	212^a^ ± 6.15 (n = 39)	<0.001

Abbreviations: GIT, gastrointestinal tract; HCP, Haylage+Corn silage+Pellet compound feed; HP, Haylage+Pellet compound feed.

a,b Different superscripts within row mark significant differences between the groups (*P* < 0.05).

**Figure 5:**
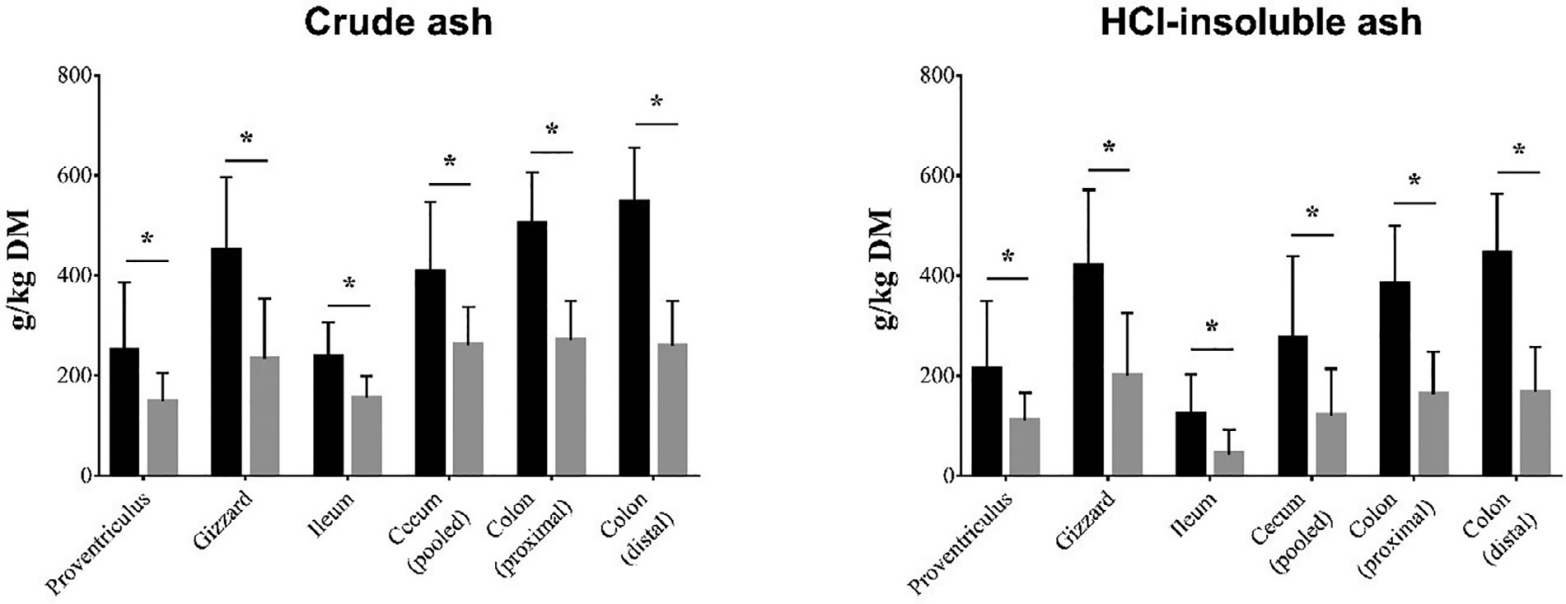
Influence of feeding on the contents of crude ash and HCl-insoluble ash in the digesta of GIT sections. The content value between the groups differs significantly (*P < 0.05). HP = Haylage+Pelleted compound feed, HCP = Haylage+Corn silage+Pelleted compound feed. Black colored columns indicate HP group, while grey colored columns indicate HCP group. Abbreviation: GIT, gastrointestinal tract.

Nevertheless, the contents of crude ash and HCl-insoluble ash in the digesta of all the GIT compartments were significantly higher for ostriches fed the HP diet in comparison to those fed the HCP diet (Figure 5; in details in Table S2). This means that in the case of the group fed the HP diet, they had the possibility to uptake more stones to help the gizzard to grind the fresh grasses as well as the haylage particles.

### Masses of Gastro- and Enteroliths

**Figure 6:**
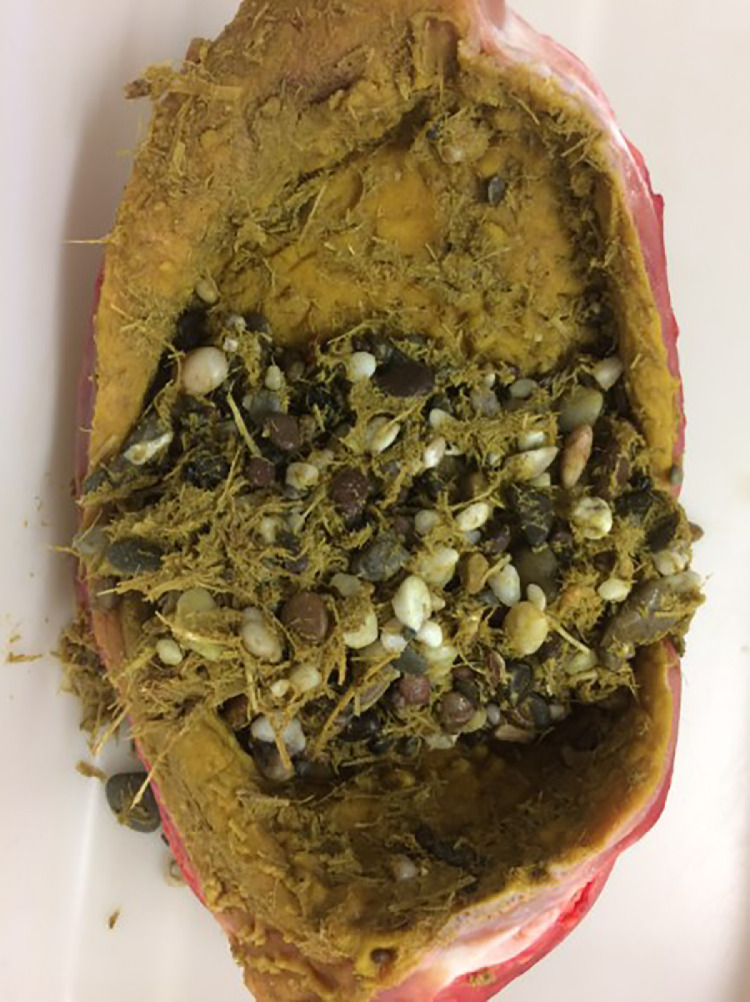
Presence of stones, pebbles in the ostrich gizzard.

In addition to the feed fed in this study, there were also stones of varying types and sizes available to the animals in the enclosure. The mass and proportion of the gastro- and enteroliths in the individual compartments of GIT are listed in Table 5. The weight of stones in the proventriculus had higher significant value (229 g) in comparison to the individual compartments in the small intestine, cecum, and colon. While, the gizzard had the highest significant stone mass (1,213 g) compared to the other individual GIT compartments. Regarding the proportion of stones, pebbles and sand in the different GIT compartments, it was observed that the percentage of stones in the gizzard was significantly the highest (4.76%) compared to the other individual organs (Figure 6). No stones were found in the small intestine, cecum or colon. Nevertheless, the distal colon had the highest significant proportion of sand (30.3%) in comparison to the other individual organs. In contrast, the lowest significant proportion of sand was recorded for jejunum (0.33%) compared to other individual organs in the GIT except for the proventriculus (6.43%) and colon (22.0 and 30.3% for proximal and distal parts, respectively). Regarding the effect of feed type on the masses of gastroliths, it was found that ostriches fed the HP diet had gastroliths weighing about 1,338 g in the gizzard vs. 1,143 g for the group fed the HCP diet (details in Table S3). Also, the masses of enteroliths in the colon (proximal and distal parts) were about 29.1 and 45.7 g in those animals fed the HP diet vs. 16.3 and 3.73 g for the group fed the HCP diet (details in Table S3).

**Table 5:** Mass and proportion of the gastro- and enteroliths of the individual compartments of the GIT (n = 61) independent of the type of feed offered.

Item	Stomach		Small intestine			Cecum		Colon		
	proventriculus	gizzard	duodenum	jejunum	ileum	left part	right part	proximal part	distal part`	*P*-value
Stone mass (g)	229^b^ ± 27.3	1213^a^ ± 52.2	0.63^d^ ± 0.26	0.16^d^ ± 0.08	0.31^d^ ± 0.17	121^c^ ± 17.0	147^c^ ± 16.0	20.9^d^ ± 4.42	18.9^d^ ± 8.37	<0.001
Proportions (%)										
Stone	2.71^b^^w^ ± 0.64	4.76^a^^w^ ± 1.34	0.00^c^^w^ ± 0.00	0.00^c^^w^ ± 0.00	0.00^c^^w^ ± 0.00	0.00^c^^w^ ± 0.00	0.00^c^^w^ ± 0.00	0.00^c^^w^ ± 0.00	0.00^c^^w^ ± 0.00	<0.001
Pebbles	90.9^a^^v^ ± 2.40	94.4^a^^v^ ± 1.80	22.2^c^^v^ ± 3.80	14.4^c^^v^ ± 3.30	20.3^c^^v^ ± 2.50	95.3^a^^v^ ± 1.80	93.9^a^^v^ ± 2.40	79.8^b^^v^ ± 4.20	69.7^b^^v^ ± 3.60	<0.001
Sand	6.43^c^^w^ ± 2.40	0.86^cd^^x^ ± 0.02	0.72^cd^^w^ ± 0.10	0.33^d^^w^ ± 0.04	0.98^cd^^w^ ± 0.27	4.71^cd^^w^ ± 1.80	4.50^cd^^w^ ± 1.80	22.0^b^^w^ ± 3.62	30.3^a^^w^ ± 3.58	<0.001
*P*-value	<0.001	<0.001	0.001	<0.001	<0.001	<0.001	<0.001	<0.001	<0.001	

Abbreviation: GIT, gastrointestinal tract.

a,b,c,d Different superscripts within row mark significant differences between the groups (*P* < 0.05).

v,w,x Different superscripts within column mark significant differences between the groups (*P* < 0.05).

Stones like those found in the proventriculus and gizzard were definitely not present in the small intestine. Under aspects of comparative nutrition, ostriches are able to utilize roughage plants as a basic food source without having teeth for mastication or processes like rumination (Milton and Dean, 1995). In the absence of teeth for mechanical comminution of plant structures, ostriches are dependent on the uptake of stones (Huchzermeyer, 1994). Previous studies show that ostriches take up stones daily in addition to feed (Holtzhausen and Kotzé, 1990; Milton and Dean, 1995). The filter-like mucous membrane folds of the stomach gate allow only the smallest particles to pass through, while larger structures are retained in the gizzard for further comminution of the feed (Klasing, 1998; Nickel et al., 2004). However, the masses of gastroliths in the stomach of the ostriches correlated neither with sex, age, season nor with the masses of feed consumed (Wings, 2004). There is clear evidence that the particle size and the physical form of the diet have an effect on the development of the birds’ digestive system, which is also accompanied by changes in physiological functions (Svihus, 2014). Moreover, it is well defined that the gizzard is a dynamic organ which consequently responds rapidly to dietary changes (Svihus, 2014). Gizzard size may increase to over 100% of its original size when structural components are added to the diet (Svihus, 2011). Svihus et al. (2004) found that feeding broiler chickens coarsely wheat led to an increased gizzard weight compared to finely wheat (17.5 vs. 14.9 g/kg BW).

### Particle Size Distribution of Gastro- and Enteroliths in the GIT

Table 6 shows the particle size distribution (in %) for gastro- and enteroliths in different individual sections of the GIT regardless of the feed type. The results revealed that the gizzard had the significantly highest proportion of particles larger than 19 mm (4.76%) compared to other GIT sections. Also, regarding the 8 mm fraction, the proventriculus and gizzard showed the significantly highest percentage (72.3 and 75.6%, respectively) in comparison to other GIT compartments. Regarding the 4 mm fraction size, the cecum (left and right) showed the significantly highest values (51.9 and 52.2%, respectively) compared to other GIT organs. The colon (proximal and distal) had the significantly highest proportion of >3.15 mm fraction (7.67 and 6.22%, respectively) compared to other individual GIT organs (except for the left cecum). The distal colon had significantly the highest proportion of the 0.8, 0.56, and 0.4 mm fractions (2.42, 4.57 and 6.22%, respectively) compared to all other individual GIT organs, whereas both the proximal and distal colon had significantly the highest proportion of 0.2 mm and <0.2 mm fractions than for other individual GIT organs.

**Table 6:** Particle size (%) of the gastro- or enterolith mixtures of the individual sections of the GIT independent of the type of feed offered.

	Stomach		Small intestine			Cecum		Colon		
Size (mm)	proventriculus	gizzard	duodenum	jejunum	ileum	left part	right part	proximal part	distal part	*P*-value
19	2.71^b^^x^ ± 0.64	4.76^a^^x^ ± 1.34	0.00^c^^x^ ± 0.00	0.00^c^^w^ ± 0.00	0.00^c^^w^ ± 0.00	0.00^c^^z^ ± 0.00	0.00^c^^y^ ± 0.00	0.00^c^^z^ ± 0.00	0.00^c^^y^± 0.00	< 0.001
8	72.3^a^^v^ ± 2.48	75.6^a^^v^± 1.83	1.66^d^^wx^ ± 0.98	2.29^d^^w^± 1.75	2.17^d^^w^ ± 1.19	30.1^b^^w^ ± 2.64	29.1^b^^w^ ± 2.07	14.7^c^^w^ ± 2.34	10.7^c^^w^±2.03	< 0.001
4	16.4^d^^w^ ± 1.41	17.3^d^^w^ ± 1.47	13.7^de^^v^ ± 3.76	8.16^e^^v^ ± 3.27	6.46^e^^v^± 2.51	51.9^a^^v^ ± 2.82	52.2^a^^v^ ± 2.37	40.9^b^^v^ ± 3.16	32.4^c^^v^±3.54	< 0.001
3.15	0.40^d^^x^ ± 0.05	0.387^d^^y^ ± 0.03	0.84^d^^wx^ ± 0.35	0.23^d^^w^ ± 0.16	0.68^d^^w^ ± 0.38	4.16^bc^^xy^ ± 1.45	2.47^cd^^y^ ± 0.25	7.67^a^^xy^ ± 2.11	6.22^ab^^x^± 1.56	< 0.001
2	1.01^e^^x^ ± 0.11	0.90^e^^y^ ± 0.08	3.81cde^w^ ± 1.25	3.03^de^^w^ ± 1.73	8.78^b^^v^ ± 2.91	7.41^bc^^x^ ± 0.81	7.19bcd^w^ ± 1.15	10.8^ab^^wx^ ± 1.47	13.3^a^^w^± 1.87	< 0.001
1.4	0.37^de^^x^ ± 0.07	0.17^e^^y^ ± 0.02	1.64^cd^^wx^ ± 0.89	0.44^de^^w^ ± 0.29	2.09^bc^^w^ ± 0.92	1.19cde^yz^ ±0.14	1.39cde^y^ ± 0.36	3.16^ab^^z^ ± 0.32	4.00^a^^xy^ ± 0.64	< 0.001
1	0.39^cd^^x^ ± 0.11	0.08^d^^y^ ± 0.02	0.58^cd^^wx^ ± 0.35	0.28^cd^^w^ ± 0.19	0.17^d^^w^ ± 0.11	0.55^cd^^z^ ± 0.08	1.51^bc^^y^ ± 1.02	2.53^ab^^z^ ± 0.74	3.09^a^^xy^ ± 0.46	< 0.001
0.8	0.40^c^^x^ ± 0.13	0.05^c^^y^ ± 0.02	0.17^c^^x^ ± 0.10	0.04^c^^w^ ± 0.04	0.11^c^^w^ ± 0.08	0.29^c^^z^ ± 0.06	0.46^c^^y^ ± 0.19	1.25^b^^z^ ± 0.15	2.42^a^^xy^ ± 0.39	< 0.001
0.56	1.78^c^^x^ ± 0.70	0.13^d^^y^ ± 0.05	0.19^d^^x^ ± 0.10	0.12^d^^w^ ± 0.08	0.16^d^^w^ ± 0.10	1.36^c^^yz^ ± 0.59	0.95^cd^^y^ ± 0.42	3.23^b^^z^ ± 0.47	4.57^a^^x^ ± 0.56	< 0.001
0.4	1.57^c^^x^ ± 0.60	0.15^c^^y^ ± 0.06	0.16^c^^x^ ± 0.09	0.12^c^^w^ ± 0.09	0.29^c^^w^ ± 0.26	1.34^c^^yz^ ± 0.77	1.27^c^^y^ ± 0.66	3.82^b^^yz^ ± 0.61	6.22^a^^x^ ± 0.92	< 0.001
0.2	2.39^b^^x^ ± 0.92	0.42^c^^y^ ± 0.15	0.15^c^^x^ ± 0.08	0.04^c^^w^ ± 0.03	0.26^c^^w^ ± 0.23	1.09^b^^yz^ ± 0.34	1.28^b^^y^ ± 0.46	9.60^a^^x^ ± 1.77	12.3^a^^w^ ± 1.68	< 0.001
<0.2	0.28^bc^^x^ ± 0.11	0.10^b^^y^ ± 0.03	0.05^b^^x^ ± 0.02	0.01^b^^w^ ± 0.01	0.16^b^^w^ ± 0.16	0.63^b^^z^ ± 0.42	0.55^b^^y^ ± 0.27	4.05^a^^yz^ ± 0.83	4.76^a^^x^ ± 0.89	< 0.001
*P-*value	<0.001	<0.001	<0.001	<0.001	<0.001	<0.001	<0.001	<0.001	<0.001	

Abbreviation: GIT, gastrointestinal tract.

a,b,c,d,e Different superscripts within row mark significant differences between the groups (*P* < 0.05).

v,w,x,y,z Different superscripts within column mark significant differences between the groups (*P* < 0.05).

Interestingly, according to the type of feed offered to the ostriches, the size of stones eaten, differed. The particle size distribution (%) of stones in the gizzard >1 mm was higher (*P* < 0.05) 1.43% for ostriches fed the HP diet vs. 0.66% for those fed the HCP diet (details in Table S4). About 1.25% of the average BW of ostriches was made up of gastroliths, which were found in both stomach sections (proventriculus, gizzard). These observations are in agreement with previous studies reporting that about 1 to 1.5 kg of stones found or about 1% of the body mass was made up of gastroliths in both stomach sections (Holtzhausen and Kotzé, 1990; Wings, 2004). The highest amount of gastroliths was found in the gizzard, where these helped to grind green fodder and roughage to a fiber length similar to that found in the GIT of cattle (Holtzhausen and Kotzé, 1990; Aganga et al., 2003). Our findings were similar to a study reporting the particle size of digesta as being significantly larger in the stomach than in the cecum or colon (Fritz et al., 2012). Generally, it could be stated that stones occurred only in the 2 stomach sections, while small stones were present in other intestinal compartments and sand dominated in the colon. Continuously consumed stones are almost completely excreted as sand. Therefore, continuous stone replacements might be necessary for ostriches to mechanically break down hard or coarse food in the gizzard.

**Figure 7:**
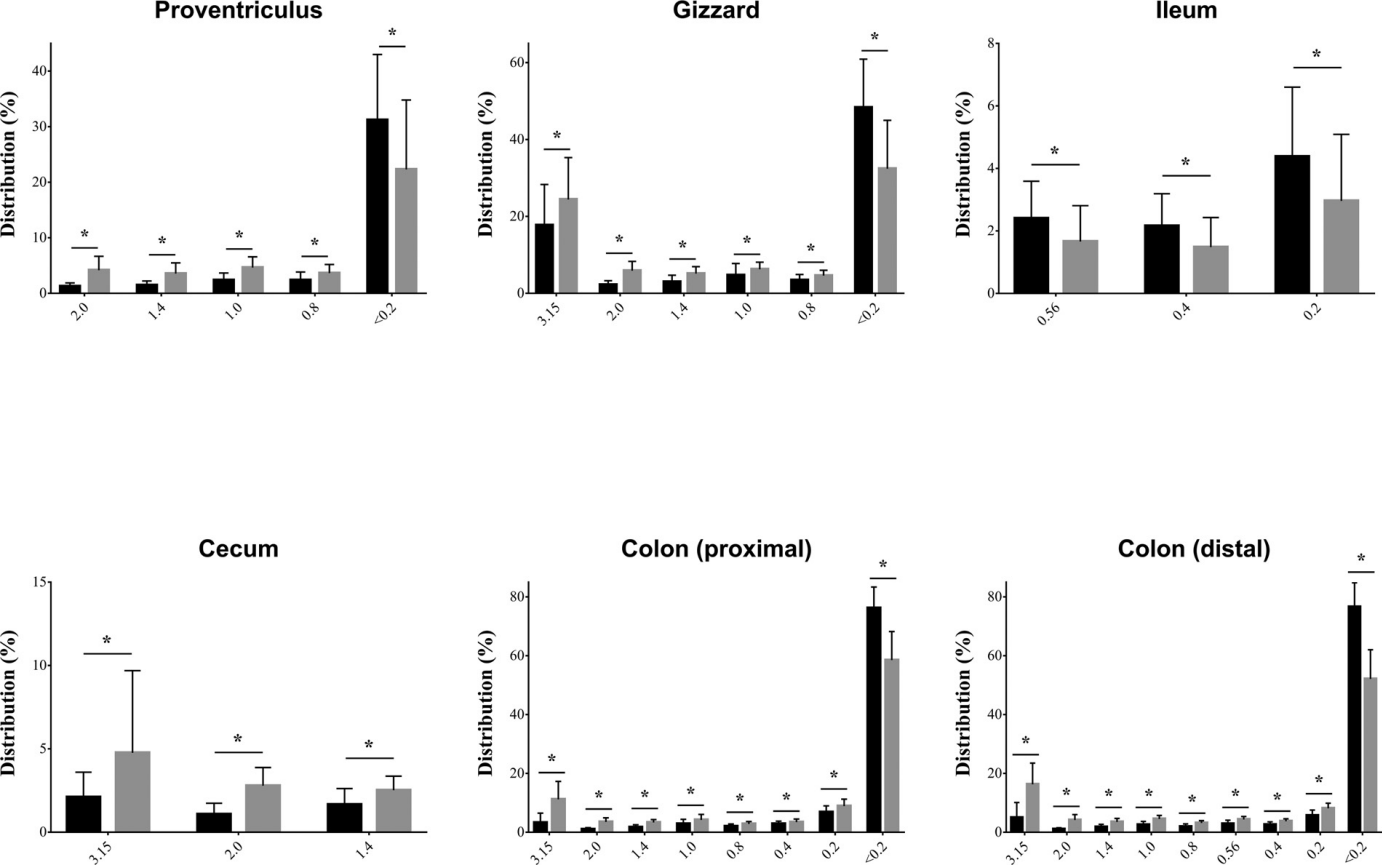
Particle size distribution (%) of the digesta of the analyzed sections of the GIT. The content values between the groups differ significantly (*P < 0.05). HP = Haylage+Pelleted compound feed, HCP = Haylage+Corn silage+Pelletede compound feed. Black colored columns indicate HP group, while grey colored columns indicate HCP group. Abbreviation: GIT, gastrointestinal tract.

The effects of feed on the particle size proportion of digesta in each GIT section are presented in Figure 7 (details in Tables S5 and S6). It was noted that ostriches fed the HCP diet had a significantly higher digesta proportion in the proventriculus of the 0.8 mm to 2 mm fraction compared to those fed the HP diet. Ostriches fed the HCP diet had a higher digesta proportion in the gizzard of the 0.8 mm to 3.15 mm fractions compared to those fed the HP diet (*P* < 0.05). Nevertheless, ostriches fed the HP diet had a higher (*P* < 0.05) digesta proportion in the ileum of the 0.2 mm to 0.56 mm fractions in comparison to those fed the HCP diet. In the cecum digesta, ostriches fed the HCP diet had a higher (*P* < 0.05) digesta proportion of the 1.4 mm to 3.15 mm fraction than those fed the HP diet. Ostriches fed the HCP diet had a higher (*P* < 0.05) digesta proportion in the colon (proximal and distal) of the 0.2 mm to 3.15 mm fraction than those fed the HP diet. Generally, ostriches fed the HP diet had a higher (*P* < 0.05) digesta proportion of the <0.2 mm fraction than those animals fed the HCP diet for the stomach (proventriculus, gizzard) and the colon (proximal, distal). Ramadhani (2000) found that ostriches possess the ability to ferment roughage and the amount of total volatile fatty acids in the hindgut content increased with increasing levels of roughage in the diet. This is not surprising considering the natural feeding habit of ostriches. Swart et al. (1993) and Hastings (1994) stated that the long retention time of fibrous feed and/or the digesta in the GIT ensures exposure of feed particles to microbial digestion for extended periods and a high concentration of volatile fatty acids.

## CONCLUSIONS

Taking the results of this study into account, increasing the feed particle size (>3.15 mm) led to an increase in the empty gizzard weight as in case of feeding HCP diet. By feeding the HP diet (with more fresh grasses in spring/summer season), the crude fiber content in the whole GIT digesta was decreased and the crude ash and HCl-insoluble ash contents increased compared to those animals fed HCP diet (low fresh grasses in fall/winter season). This means that increasing the dietary fiber content and/or its particle size led to the ostriches eating more stones of a large size. Stones were found in all GIT sections, while sand was present only in the colon. As a result of this “cascade”, stones in the stomachs, pebbles, and sand in the intestinal tract, it can be concluded that the stones ingested were crushed as a result of abrasive processes in the gizzard and finally excreted as smaller pebbles and sand. This means that with the absence of teeth (mechanical grinding) in ostriches, there is a need for a substitute enabling the birds to comminute the green fodder and roughage.

## Appendix Supplementary materials

Image, application 1
