# Supplementary material for: Studies on the weight of the gastrointestinal tract, digesta composition and occurrence of gastro- and enteroliths in adult domesticated ostriches fed different diets
Source: Poult Sci. 2021 Jun 26;100(9):101359. doi: 10.1016/j.psj.2021.101359 (PMC8342792; doi:10.1016/j.psj.2021.101359)
Supplement: Supplementary file 1 [file mmc1.docx]

**SUPPLEMENTARY DATA**

**Table S1.** Influence of the different feedings on the dimensions (cm) of the small intestine sections.

| Group | Duodenum | Jejunum | Ileum |
| --- | --- | --- | --- |
| HP | 126^b^±11.1 | 207^a^±27.7 | 486^a^±61.3 |
| HCP | 132^a^±11.8 | 221^a^±32.9 | 478^a^±70.9 |

^a,b^ Different superscripts within column mark signiﬁcant differences between the groups (*P* < 0.05). HP = Haylage+Pellet compound feed (n = 22), HCP = Haylage+Corn silage+Pellet compound feed (n = 39).

**Table S2.** The crude ash and HCl-insoluble ash contents in the GIT digesta of ostriches fed different diets.

| Parameter  (g/kg DM) | Organ | HP | HCP |
| --- | --- | --- | --- |
| Crude ash | Proventriculus | 251^a^±136  (n=20) | 149^b^±57.3  (n=32) |
|  | Gizzard | 450^a^±146  (n=20) | 233^b^±121  (n=36) |
|  | Ileum | 238^a^±69.1  (n=22) | 156^b^±43.5  (n=38) |
|  | Cecum (pooled) | 408^a^±139  (n=21) | 261^b^±76.8  (n=32) |
|  | Colon (proximal) | 505^a^±102  (n=22) | 272^b^±77.5  (n=39) |
|  | Colon (distal) | 547^a^±109  (n=22) | 259^b^±90.0  (n=39) |
| HCl-insoluble ash | Proventriculus | 215^a^±134  (n=20) | 112^b^±54.9  (n=32) |
|  | Gizzard | 420^a^±152  (n=20) | 201^b^±124  (n=36) |
|  | Ileum | 123^a^±79.8  (n=22) | 44.2^b^±48.8  (n=38) |
|  | Cecum (pooled) | 276^a^±163  (n=21) | 122^b^±92.1  (n=32) |
|  | Colon (proximal) | 384^a^±116  (n=22) | 164^b^±84.5  (n=39) |
|  | Colon (distal) | 445^a^±119  (n=22) | 168^b^±89.2  (n=39) |

^a,b^ Different superscripts within row mark signiﬁcant differences between the groups (*P* < 0.05). HP = Haylage+Pellet compound feed, HCP = Haylage+Corn silage+Pellet compound feed.

**Table S3.** Mass (g) of the gastro- and enteroliths of the individual compartments of the GIT based on the type of feed offered.

| Organ | HP | HCP |
| --- | --- | --- |
| Proventriculus | 164^b^±140 | 265^a^±239 |
| Gizzard | 1338±356 | 1143±423 |
| Duodenum | 0.94±2.29 | 0.45±1.81 |
| Jejunum | 0.03^b^±0.13 | 0.24^a^±0.72 |
| Ileum | 0.67^a^±2.16 | 0.10^b^±0.37 |
| Cecum (left) | 97.3±127 | 134±135 |
| Cecum (right) | 122±114 | 161±131 |
| Collon (proximal) | 29.1±41.6 | 16.3±29.5 |
| Colon (distal) | 45.7^a^±104 | 3.73^b^±8.64 |

^a,b^ Different superscripts within row mark signiﬁcant differences between the groups (*P* < 0.05). HP = Haylage+Pellet compound feed, HCP = Haylage+Corn silage+Pellet compound feed.

**Table S4.** Particle size distribution (%) of the stones in gizzard based on the type of feed offered.

| Particle size | HP (n=22) | HCP (n=39) |
| --- | --- | --- |
| > 1mm | 1.43^a^±0.77 | 0.66^b^±1.56 |
| < 0.2mm | 2.77^a^±4.49 | 0.46^b^±1.42 |

^a,b^ Different superscripts within row mark signiﬁcant differences between the groups (*P* < 0.05). HP = Haylage+Pellet compound feed, HCP = Haylage+Corn silage+Pellet compound feed.

**Table S5.** Particle size distribution (%) of the digesta of the analyzed sections of the GIT.

| Organ | Fraction, mm | HP | HCP |
| --- | --- | --- | --- |
| Proventriculus | 2 | 1.26^b^±0.61 | 4.15^a^±2.52 |
|  | 1.4 | 1.47^b^±0.76 | 3.56^a^±1.92 |
|  | 1 | 2.38^b^±1.29 | 4.63^a^±1.94 |
|  | 0.8 | 2.36^b^±1.48 | 3.63^a^±1.57 |
|  | < 0.2 | 31.2^a^±11.8 | 22.3^b^±12.5 |
| Gizzard | 3.15 | 17.7^b^±10.6 | 24.4^a^±10.9 |
|  | 2 | 2.23^b^±1.05 | 5.89^a^±2.42 |
|  | 1.4 | 2.98^b^±1.73 | 5.13^a^±1.81 |
|  | 1 | 4.73^b^±3.02 | 6.26^a^±1.84 |
|  | 0.8 | 3.42^b^±1.49 | 4.60^a^±1.39 |
|  | < 0.2 | 48.3^a^±12.6 | 32.4^b^±12.6 |
| Ileum | 0.56 | 2.40^a^±1.19 | 1.66^b^±1.15 |
|  | 0.4 | 2.16^a^±1.03 | 1.48^b^±0.95 |
|  | 0.2 | 4.38^a^±2.22 | 2.96^b^±2.13 |
| Cecum (pooled) | 3.15 | 2.10^b^±1.50 | 4.76^a^±4.93 |
|  | 2 | 1.08^b^±0.66 | 2.79^a^±1.09 |
|  | 1.4 | 1.66^b^±0.96 | 2.52^a^±0.84 |

^a,b^ Different superscripts within row mark signiﬁcant differences between the groups (*P* < 0.05). HP = Haylage+Pellet compound feed, HCP = Haylage+Corn silage+Pellet compound feed. Number of measurements for HP and HCP in proventriculus (n = 19 and 34), gizzard (n = 21 and 36), ileum (n = 22 and 33), cecum (n = 21 and 30), respectively.

**Table S6.** Particle size distribution (%) of the digesta of the analyzed sections of the GIT.

| Organ | Fraction, mm | HP | HCP |
| --- | --- | --- | --- |
| Colon (proximal) | 3.15 | 3.28^b^±3.22 | 11.2^a^±6.08 |
|  | 2 | 1.03^b^±0.55 | 3.50^a^±1.43 |
|  | 1.4 | 1.73^b^±0.84 | 3.34^a^±0.97 |
|  | 1 | 2.87^b^±1.55 | 4.20^a^±1.88 |
|  | 0.8 | 2.05^b^±0.78 | 2.83^a^±0.83 |
|  | 0.4 | 2.86^b^±0.93 | 3.46^a^±1.03 |
|  | 0.2 | 6.84^b^±2.13 | 8.90^a^±2.34 |
|  | < 0.2 | 76.2^a^±7.13 | 58.5^b^±9.73 |
| Colon (distal) | 3.15 | 4.94^b^±5.18 | 16.3^a^±7.20 |
|  | 2 | 1.06^b^±0.47 | 4.18^a^±1.85 |
|  | 1.4 | 1.82^b^±0.88 | 3.52^a^±1.19 |
|  | 1 | 2.57^b^±1.11 | 4.53^a^±1.18 |
|  | 0.8 | 1.92^b^±0.92 | 3.14^a^±0.83 |
|  | 0.56 | 2.89^b^±1.21 | 4.35^a^±1.01 |
|  | 0.4 | 2.59^b^±0.95 | 3.76^a^±0.83 |
|  | 0.2 | 5.70^b^±1.86 | 8.15^a^±1.74 |
|  | < 0.2 | 76.6^a^±8.21 | 52.1^b^±9.92 |

^a,b^ Different superscripts within row mark signiﬁcant differences between the groups (*P* < 0.05). HP = Haylage+Pellet compound feed, HCP = Haylage+Corn silage+Pellet compound feed. Number of measurements for HP and HCP in colon (n = 22 and 39), respectively.
